# Cytotoxicity and Genotoxicity Effects of a Magnetic Zeolite Composite in *Daphnia magna* (Straus, 1820)

**DOI:** 10.3390/ijms25147542

**Published:** 2024-07-09

**Authors:** Jacquelyne Y. Zarria-Romero, Juan A. Ramos-Guivar

**Affiliations:** Grupo de Investigación de Nanotecnología Aplicada para Biorremediación Ambiental, Energía, Biomedicina y Agricultura (NANOTECH), Facultad de Ciencias Físicas, Universidad Nacional Mayor de San Marcos, Av. Venezuela Cdra 34 S/n, Ciudad Universitaria, Lima 15081, Peru; jacquelyne.zarria@unmsm.edu.pe

**Keywords:** Cladocera, short-term acute assay, genotoxicity profile, composite ecotoxicity, zeolite 5A, magnetic nanoparticles

## Abstract

Zeolite type 5A combined with the magnetic properties of maghemite nanoparticles facilitate the rapid absorption of heavy metals, which makes them an interesting proposal for the remediation of water contaminated with lead and arsenic. However, the physicochemical analysis related to concentration and size for the use of this magnetic zeolite composite (MZ0) in water bodies and the possible toxicological effects on aquatic fauna has not yet been carried out. The main objective of the research work is to determine lethal concentrations that cause damage to *Daphnia magna* based on *LC*_50_ tests, morphology, reproductive rate, and quantification of the expression of three genes closely involved in the morphological development of vital structures (*Glass*, *NinaE*, *Pph13*). To achieve this objective, populations of neonates and young individuals were used, and results showed that the *LC*_50_ for neonates was 11,314 mg L^−1^, while for young individuals, it was 0.0310 mg L^−1^. Damage to morphological development was evidenced by a decrease in eye size in neonates, an increase in eye size in young individuals, variations in the size of the caudal spine for both age groups, and slight increases in the heart size, body, and antenna for both age groups. The reproductive rate of neonates was not affected by the lower concentrations of MZ0, while in young individuals, the reproductive rate decreased by more than 50% from the minimum exposure concentration of MZ0. And for both ages, *Glass* gene expression levels decreased as the MZ0 concentration increased. Also, the MZ0 evidenced its affinity for the exoskeleton of *D. magna*, which was observed using both light microscopy and electron microscopy. It is concluded that MZ0 did not generate significant damage in the mortality, morphology, reproductive rate, or gene expression in *D. magna* at lower concentrations, demonstrating the importance of evaluating the possible impacts on different life stages of the cladoceran.

## 1. Introduction

Nanoparticles (NPs) are widely used in various areas of science due to their handling and control physicochemical characteristics [1]. For example, titanium dioxide (TiO_2_) NPs have demonstrated utility as a bactericide [2]. Adsorbent properties regarding magnetic nanohybrids/nanocomposites or composites are compelling materials useful for water remediation due to their chemical affinity, surface response, specific surface area, and magnetism [3]. However, the evidence of the compatibility of nanomaterials (NMs) with the ecosystem is limited despite significant investments in nanotechnology research [4,5]. Moreover, with the raising of micro(nano)plastics contamination, there is huge concern about permissible concentrations for NMs [6].

Although NPs can damage living beings at certain doses, they also have the potential to aid in wastewater remediation or water bodies recuperation [7,8]. For these reasons, it is crucial to understand the ecotoxicological properties of adsorbents, in different biomarkers [9,10], used in the remediation of contaminated effluents, as their massive use can have negative impacts on aquatic life. Two scenarios are expected: (i) in a first stage, the NMs will spread from industrial water into the environment, affecting the population dynamics, and hence the long-term effects need to be studied, (ii) the NMs are used in-situ as fast adsorbents to remediate a water body polluted with certain pollutant; this process occurs in a few hours, affecting the heterogenous population, since a single NMs concentration can have toxic effects on aquatic organisms; therefore, short-term acute effects should be evaluated to study the lethal dose concentration and probable morphological alteration caused by NMs.

The results of these studies can be used to support the regulation of NMs and their use in industry to minimize their impact on the environment as well as their water recovery applications [11]. In our previous work, we have studied the Pb adsorption properties presented in real water using the MZ0 sample. Up to 100% Pb removal from real effluents was achieved using 1 g L^−1^ of adsorbent dose [12]. Nevertheless, this concentration must agree with the ecotoxicity profile to have a complete environmental analysis.

The present work focuses on the first study investigating the ecotoxicological effects of the MZ0 hybrid on *D. magna*, because magnetic zeolite adsorbents have demonstrated a high removal capacity of heavy metals from aquatic ecosystems, but their toxicity toward aquatic species has not yet been investigated yet. For that purpose, a standardized short-term acute assay was developed to find the 24 h lethal concentration (24 h-*LC*_50_) and to study the morphological damage after exposure, the reproduction rate, and the genotoxicity effects. Therefore, this research attempts to fill this gap in knowledge and provide crucial information for the formulation of policies for the safe use of these NMs, NPs, nanocomposites, or hybrids made of composite plus NMs.

## 2. Results and Discussion

### 2.1. LC_50_ Analysis

Neonates and young individuals were exposed to MZ0 concentrations of 25, 62.5, 125, 250, and 500 mg L^−1^ and the negative control (N.C.) without alteration in the medium. Mortality percentage was observed differently for both populations of individuals that differed in age.

**Figure 1:**
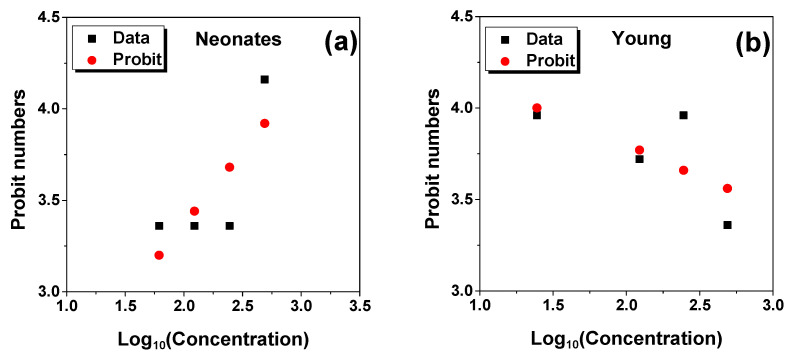
Probit numbers vs. concentration logarithm. In (**a**), the plot indicates the Probit linear regression analysis for neonate individuals exposed to the MZ0 sample. In (**b**), the plot illustrates the Probit analysis for young individuals exposed to the MZ0 sample.

Figure 1a,b show the Probit linear regression plots for neonates and young individuals. It was found that the *LC*_50_ values was 11,314 mg L^−1^ and 0.0310 mg L^−1^ for neonates and young individuals, respectively, indicating that young individuals are more sensitive than newborns to the MZ0 hybrid. The *LC*_50_ value found for MZ0 agrees with that reported for Fe_3_ O_4_ NPs (4965.92 mg L^−1^) [13]. It was observed to decrease with exposure time. 

Table 1 summarizes the *LC*_50_ values found for other NMs in *D. magna* and other aquatic species. It is observed that the exposure time is a key parameter for *LC*_50_ determination. Ecotoxicity properties of zeolites are lacking in the literature. In this study, we prioritized the ecotoxicological profile of the magnetic composite MZ0, since zeolite by itself cannot be magnetically manipulated, and the MZ0 sample is superior for magnetic remediation. Nanotoxicology is an emerging area, and most of the research has focused on individual NMs with different size properties. However, some nanocomposites or composites have been evaluated in *D. magna*. For example, six reduced graphene oxide (rGO) nanocomposites (rGO-Au, rGO-Ag, rGO-Pd, rGO-Fe_3_O_4_, rGO-Co_3_O_4_ and rGO-SnO_2_; approximately 0.5–5 mm in lateral size) were evaluated in *D. magna* neonates (<24 h old) [14]. Among them, 100% of deaths were caused by rGO-Ag in the first 24 h, where Ag was found to be acutely toxic and susceptible of affecting *D. magna* juveniles. In that sense, NMs can diffuse more rapidly due to their rapid uptake constant. In addition, the range of concentrations in that study was 0.02 to 0.12 mg L^−1^, hence indicating that individual NMs have higher toxicity. Natural material like pyroclastic dust did not lead to mortality in *D. magna*, but after modifying their surface with iron oxide NPs (24.1 wt%), a value of *LC*_50_ equal to 123.6 mg L^−1^ was found in 24 h *D. magna* neonates [15]. In general, pure iron oxide NPs or magnetic composites present highly variable ecotoxicological response depending on the size, morphology, composition, and age of the exposed individuals. Regarding this last aspect, juvenile *D. magna* individuals exposed during 24 h to nanocomposites of MWCNTs-γ-Fe_2_O_3_ NMs and GO- γ-Fe_2_O_3_ NMs reported *LC*_50_ values of 381.8 and 0.9 mg L^−1^ [16].

In terms of chemical composition, some NMs are more toxic than others. Ag NPs have been shown to be more toxic than Au or Si NPs [26]. Furthermore, the NMs shape can also influence its toxicity. For example, MWCNTs have been shown to be more toxic than SWCNTs [27].

On the other hand, ultrasmall NMs have shown greater toxicity than larger NMs, because smaller NMs can penetrate more easily into the cells and tissues of organisms, which increases their potential to be toxic [26]. In addition, the NMs’ size may have also influenced their ability to bind proteins and other molecules in the aquatic environment, which in turn can affect its toxicity [28].

In short, the magnetic composite MZ0 presents a low toxic response to *D. magna* neonates, which aligns with the aim of fast contaminants removal and a less acute response.

### 2.2. Morphological Evaluation Analysis

It is important to analyze the NPs effects on newborn Daphnia because these organisms represent the key species in the aquatic food chain and, therefore, their health and survival rate are crucial for the stability of the water ecosystem [29]. Moreover, newborns of Daphnia are more vulnerable to the toxic effects of pollutants or NMs than juvenile and adult individuals [30]. It is worth mentioning that *Daphnia* neonates have a developing immune and nervous system, which can make them more susceptible to the toxic effects of NMs [31]. In addition, newborns of *Daphnia* have a larger body surface area in relation to their volume, which increases their rate of pollutant absorption compared to adult or juvenile individuals [32].

Although chronic exposure to NMs can provide valuable information about the long-term effects, it is also important to consider the short-term impacts of a single exposure in both newborns and juvenile or adult individuals. For example, the exposure of TiO_2_ NPs caused oxidative stress and decreased acetylcholinesterase activity in the oysters muscle [33], while the exposure for 24 h to Ag NPs caused an increase in mortality and a decrease in the food activity of marine worms [34] as well as a decrease in the mobility and growth rate of zebra fish larvae, and these effects persisted up to 15 days after exposure [34]. Exposure to NMs for only 24 h can have immediate toxic effects on aquatic organisms; therefore, it is mandatory to evaluate the long-term effects after acute exposure because the toxic effect can persist in organisms for a long period after initial exposure.

**Figure 2:**
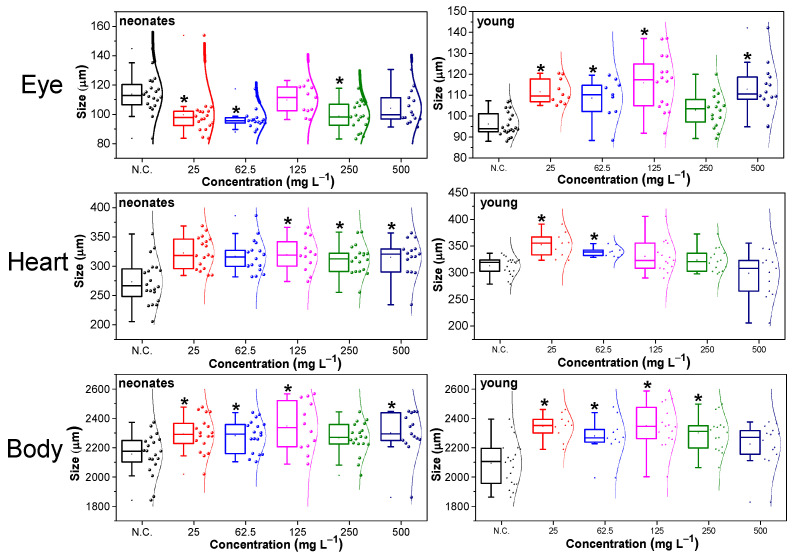
Box plots of the measurements obtained for eye, heart, and body for each of the analyzed concentrations, for neonate individuals (**left column**) as well as for juvenile individuals (**right column**). (*) indicates significance (*p* value < 0.05).

**Figure 3:**
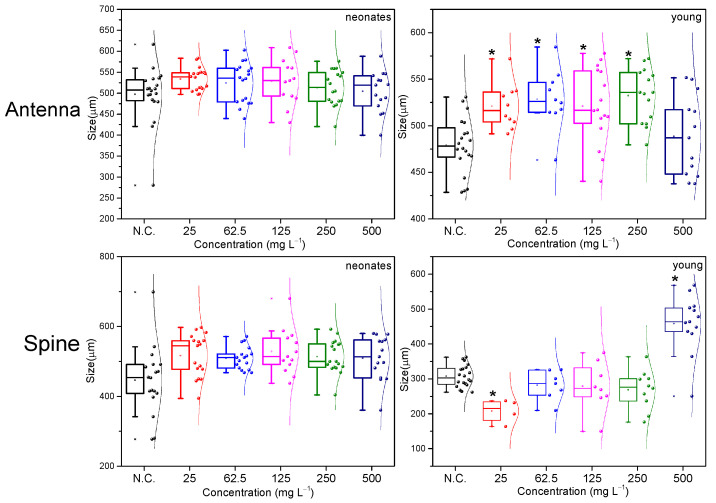
Box plots of the measurements obtained for antenna and spine for each of the analyzed concentrations, for neonate individuals (**left column**) as well as for juvenile individuals (**right column**). (*) indicates significance (*p* value < 0.05).

Figure 2 and Figure 3 showed the box plots of morphological parameters evaluated for the tested concentrations in neonate and young individuals. In Figure 2, the eye morphology was more affected in young than in neonates. In both cases, an increase or decrease in the eye size with respect to the N.C. was observed. The eye development in *D. magna* is an important parameter for evaluating the toxic effects of NMs on aquatic organisms. The exposure to NMs such as Ag, TiO_2_, and zinc oxide (ZnO) NPs has caused a decrease in the development and functionality of the composite eye in *Daphnia*, which in turn can affect its ability to detect and respond to visual stimuli in its environment [22,35,36]. 

A decrease in the number of omatidias and a reduction in the size and shape of the eyes were also observed [22,35].

*Daphnia* neonates may be more susceptible to the toxic effects of NPs because they are in an early stage of development and have not yet fully developed their immune system and other defense mechanisms. On the other hand, young *Daphnia* have a more developed immune system and may be more resistant to the NPs’ toxic effects [18,37]. 

As for the development of the eye of *D. magna*, it is known that this process occurs rapidly during the early stages of the life of the neonate; in just a few hours after echoing, the eyes of the *daphnia* begin to develop and reach their full size in approximately 24 h [38]. Therefore, the exposure to NPs during this critical stage of development could have negative effects on the growth of *D. magna*’s eye depending on the NMs concentration and exposure time [22].

The toxicity of the NMs in the heart of the genus *Daphnia* has been extensively studied; it has been shown that exposure to nanomaterial, such as TiO_2_ and Ag NPs, can cause alterations in the heart rate and amplitude of cardiac contraction in *D. magna* [39,40]. In addition, it has been shown that exposure to NMs can also induce oxidative stress in *Daphnia*, which can have a negative impact on heart function, due to the direct interaction of the NMs with heart proteins and enzymes, interfering with cellular signaling and achieving the generation of reactive oxygen species (ROS) that cause oxidational stress in the heart of *Daphnia* [41]. All this evidence underlines the importance of understanding the toxicological effects of NMs in the heart of *Daphnia* and their potential impact on the health of aquatic ecosystems [39,40,41]. In Figure 2, it was observed that neonates are more susceptible to heart size changes than young individuals. It can be noted that the average measurements for the five concentrations tested in *D. magna* are above N.C. in both neonates and young individuals, which evidences heart hypertrophy for all concentrations.

However, it is important to note that the effects of heart size may vary depending on the NMs type, the exposure concentration, and the duration of exposure. Some studies also show contradictory effects on the size of the heart of *Daphnia* exposed to NMs; it has been observed that exposure to Ag and ZnO NPs can cause a decrease in the heart size of *D. magna* [40,42], indicating that the toxicity of the NMs in the cardiac function of *Daphnia* is a complex issue that still requires further research to fully understand the underlying mechanisms.

Figure 2 depicts the box plots for the body parameter under the tested concentrations. With respect to N.C., the body parameter has increased for the neonates and young individuals. It has been reported that the atypical alteration of the body size can affect its ability to evade predators, compete with other organisms or resist parasite infection [43,44]. Similarly, alterations in the body size of *D. magna* may affect the reproduction rate, egg production, and the viability of the offspring, which could have consequences for the population and the live dynamics of the aquatic community [45,46]. As previously mentioned, *D. magna* plays an important role in the transfer of energy and nutrients in aquatic ecosystems, as it feeds on microorganisms and is an important source of food for other aquatic organisms. If its body size results are affected, it could have implications for its efficiency to transfer energy and nutrition in the trophic network, which could affect the structure and functioning of the ecosystem [43,47].

Previous studies have shown that NMs can have direct toxic effects on *D. magna*, including effects on the growth rate and body size. For example, certain types of NPs, such as Ag and ZnO, can inhibit the growth rate and reduce the body size of *D. magna* after long-term exposure [48,49]. Furthermore, some studies have indicated that the toxic effects of NMs in *D. magna* may be related to the accumulation of NMs in the body tissues, which could affect their metabolism and development [34,50].

In Figure 3, we note that the variation in the antenna size for *D. magna* is not significant for neonates, since the size averages are all almost homogeneous when comparing the treatments with the N.C. This means that there is no damage in the development of their activities or interactions with the environment or other organisms. In contrast, for young individuals (Figure 3), the individuals exposed to the highest concentration showed a slight increase in the antenna size.

NMs have been shown to affect body size in *D. magna*; one study found that exposure to SWCNTs caused a decrease in the size of the *Daphnia* body after 21 days of exposure [51]. Another study found that exposure to TiO_2_ NPs caused the interruption of molting [52]. These findings indicate that NMs may have significant effects on the morphology of the body in *D. magna*, which could have implications for their behavior and ability to interact with the aquatic environment.

Figure 3 contains the box plots for spine exposed to different concentrations. The flow spine is a structure of a polymorphic nature [53]; that is, its size varies according to the stimuli of the environment. Due to the above-mentioned polymorphism, this structure is not of high vital importance; they are usually very variable and easily alterable. However, it can be observed that in the young stage, the flow spine is more affected, which is probably because as an already developed individual, the NMs attached to the flow specimen caused an alteration in its maturation.

### 2.3. Reproductive Rate

The number of individuals obtained per litter in *D. magna* varies according to the stimuli of the environment, water quality, nutrients, and populations present in the environment of this small animal. Various pollutants alter the behavior, swimming, development, and reproduction of *D. magna* [54]. It is also worth mentioning that the age and time of maturity of the individuals is very important to obtain a good quantity of individuals by litter.

**Figure 4:**
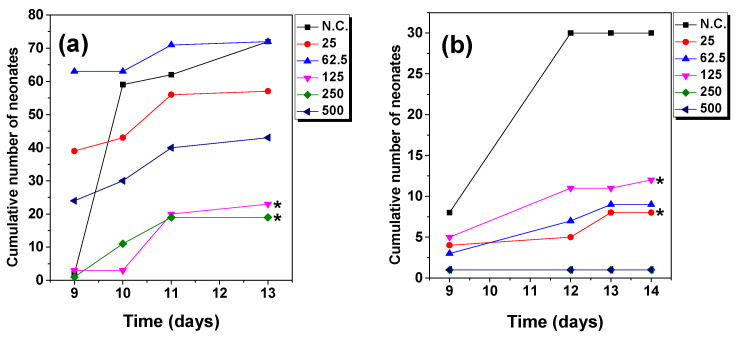
In (**a**), we observe the accumulated number of neonates obtained from individuals subjected to different concentrations of MZ0 from birth. In (**b**), we observe the accumulated number of neonates obtained from individuals subjected to different concentrations of MZ0 from their third day of life. The numbers on the legends are concentrations in mg L^−1^. (*) indicates significance (*p* value < 0.05).

Figure 4a shows the cumulative number of newborns obtained after exposing their parents to various concentrations of MZ0 within their 24 h of life; that is, after having exposed 20 neonates to five different concentrations for 24 h, these were transferred to a new clean crop to count for the next 15 days the number of offspring that can be obtained and compared with the N.C. regarding the effects of this initial exposure. In the case of N.C., this reaches a count of 70 newborns on day 13, which is in accordance with the biological reproductive cycle of *D. magna* [55]. However, after exposure, it was observed that for the two maximum concentrations of 250 and 500 mg L^−1^, the number of offspring after 15 days of monitoring did not reach even 30% of the N.C., but the lower exposure concentrations of 25 and 62.5 mg L^−1^ did not cause any significant variation to the amount of accumulated offsprings in 15 days.

In Figure 4b, young individuals exposed for 24 h to MZ0 present a serious alteration in the number of offspring. For all the tested concentrations, the accumulated newborns did not reach even the 30% with respect to the N.C., which confirmed that the age of exposure of the living organism matter [54]. These young individuals were exposed close to their reproductive age, which due to their aging did not allow them to recover as in the case of the newborn, who at lower concentrations of exposure showed a normal count of individuals.

In general, younger individuals are more susceptible than adults to environmental contaminants [56]. However, in this study, young daphnids are more sensitive than neonates. As discussed in the previous subsections, this behavior can be due to the variable heterogenous response of the MZ0 sample characterized by their unique particle size distribution and composition (composite of both bulk and nano phases). It means that this MZ0 sample cannot be analyzed as a conventional material; further experiments need to be conducted in other young and older aquatic species to prove and support their global toxic response.

### 2.4. Gene Expression

RNA was extracted and cDNA was synthesized using retrotranscription. Gene amplification by PCR was performed to analyze the band intensity based on the gene expression of three genes (*Glass*, *NinaE*, *Pph13*) that influence different parts and stages of *Daphnia*’s embryonic development from neonatal to juvenile stages. 

The *Glass*, *NinaE*, and *Pph13* genes are crucial for the development of various body parts, including the eye’s ocelos, sexual maturity, and foot development. Any changes or reduction in the expression of this gene can lead to severe consequences in the morphological development of organisms. The RNA was taken from a population of daphnids to quantify the expression level of the three genes to be examined, as the MZ0 exposure is expected within 24 h.

**Figure 5:**
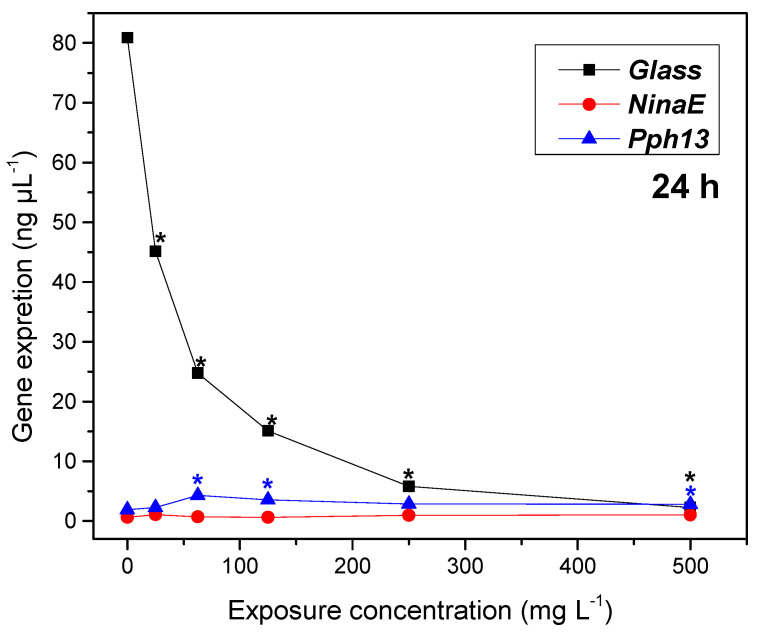
The variation in gene expression of the *Glass*, *NinaE*, and *Pph13* genes is observed according to the concentration of MZ0 to which they were exposed, 0 (N.C.), 25, 62.5, 125, 250, and 500 mg L^−1^, for exposures of 24 h. The asterisks (*) show the alteration in gene expression, which is significant compared to the N.C. by Tukey test (*p* value < 0.05).

Figure 5 shows that the gene expression of *NinaE* and *Pph13* remained stable across different concentrations, while the gene expression of *Glass* decreased as the concentration increased. In particular, the *Glass* gene exhibited a dose-dependent effect, as shown in Figure 5, after 24 h. Conversely, the *NinaE* and *Pph13* genes did not show significant differences across any of the treatments. The variability in the influence on the expression of these genes may stem from the diverse roles exhibited by the *Glass* gene throughout the entire body of the animal, making it the gene with the highest expression, whereas *NinaE* and *Pph13* have more specialized functions.

### 2.5. Interaction of MZ0 with D. magna

Currently, there are few studies that specifically investigate *Daphnia* hatching with NMs. Most studies focus on *Daphnia*’s exposure to NMs and their effects on organism survival, reproduction, and behavior. However, there is some research suggesting that exposure to NMs may affect the hatching process in *Daphnia*; the exposure of ZnO and TiO_2_ NPs caused a reduction in the hatching frequency in *D. magna* [22]. Another study found that exposure to TiO_2_ and Ag NMs interrupted the hatching process in *Daphnia* [57,58]. 

**Figure 6:**
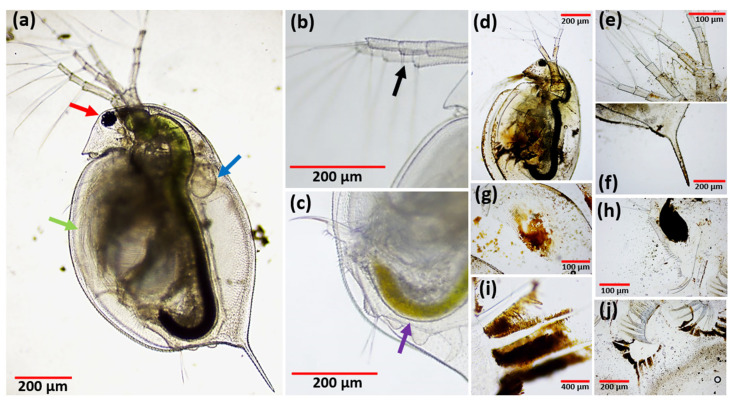
Optical images of individuals from the N.C. (**a**–**c**) where the red arrow indicates the individual’s eye, the blue arrow indicates the heart, the green arrow indicates the gills, the black arrow indicates the antennae, and the purple arrow indicates the intestine. Meanwhile, (**d**–**j**) show parts of the daphnids exposed to MZ0, where (**e**) shows the antennae, (**f**) the caudal spine, (**g**) the body valve, (**h**) the postabdomen with the last portion of the intestine, (**i**,**j**) the gills.

The exoskeleton of *Daphnia* is composed mainly of chitin; it is a carbohydrate (*n*-Acetylglucosamine) that forms part of the cell walls of different organisms as well as the resistant exoskeleton of the arthropods. The microstructure of the exoskeleton of *Daphnia* is complex and consists of multiple layers that vary in thickness and chemical composition [59,60]. In general, the exoskeleton of *Daphnia* consists of an outer layer of chitin that is covered by a thin coating of cuticular wax. Below this layer, there is a layer of proteins that act as a glue and help to bind the chitin layers. The inner layer of the exoskeleton is also composed mainly of chitin and is the thickest and strongest layer [60]. Figure 6a–c show the exoskeleton for N.C., while Figure 6d–j depict the deposition of the MZ0 sample onto the exoskeleton of daphnids.

**Figure 7:**
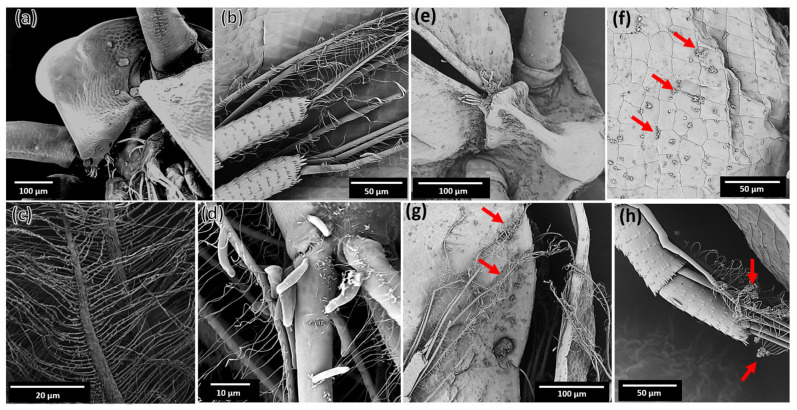
SEM images obtained by electron microscopy of individuals from the N.C. (**a**–**d**) and individuals exposed to MZ0 showing the nanohybrids adhered to their surface (**e**–**h**) indicated by red arrows.

The exoskeleton of *Daphnia* also contains minerals such as calcium, magnesium, and phosphorus, which are incorporated into the structure of the exoskeleton during the hatching process. *Daphnia* has no scales, but its exoskeleton is formed by multiple layers of chitin and proteins that can form micro-scales on its surface, as seen in Figure 7b, which is an image obtained by electron microscopy. These micro-scales are arranged in specific patterns and can provide additional protection against predators and other environmental factors [59,60,61].

**Figure 8:**
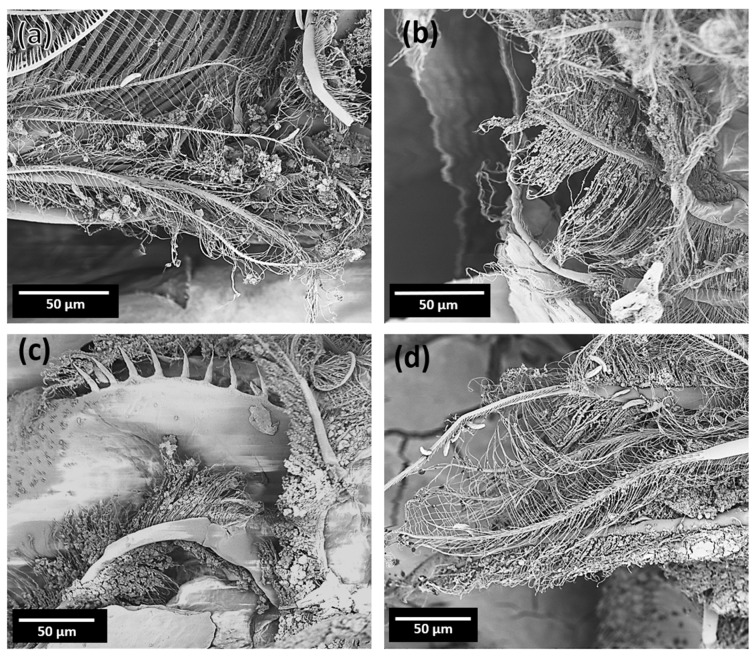
(**a**–**d**). Internal structures of the daphnids (gills) with MZ0 adhered to their surface.

The electron microscopy analysis (Figure 7 and Figure 8) allows a detailed observation of micro-structures such as scales where we can compare how the N.C. is observed (Figure 7) compared to the microphotographs obtained from *D. magna* after exposure to the MZ0 sample. According to the previous literature, parent Ag NPs cause deformations in the scales of the exoskeleton, as well as the accumulation of the particles on the surface of the body of *Daphnia* [57,62]. These results suggest that MZ0 may affect the structure of the exoskeleton of *Daphnia*, which may have consequences for its survival and function in aquatic ecosystems.

Previous studies have investigated the effects of iron oxide NPs on the morphology of the genus *Daphnia*, where exposure to iron oxide NPs affected the size and shape of the body and antennas of *D. magna* [63]. In addition, they observed changes in the structure of *Daphnia* intestines after exposure to iron oxide NPs [64] as well as disturbances in the swimming and filtration capacity of the *Daphnia* following exposure [65]. Together, these findings suggest that iron oxide NPs may have significant effects on the morphology and function of *Daphnia*, which could have implications for aquatic ecosystems.

On the other hand, some studies have also investigated the effects of zeolite NPs on *Daphnia*. For example, in prolonged exposures, they observed that exposure to zeolite NPs modified the size and shape of *Daphnia*’s body as well as its growth and survival rate [66]. These results suggest that zeolite NPs may have significant effects on the morphology, growth, and immune response of *Daphnia*. Together, these studies highlight the need to continue investigating the effects of NPs on aquatic organisms and aquatic ecosystems in general as well as taking into account exposure times.

After only 24 h of exposure to an MZ0 sample of 250 mg L^−1^, the *Daphnia* individuals did not present high rates of mortality or major morphological changes. The microphotographs in Figure 7 and Figure 8 show the interaction of the MZ0 sample with the surfaces of the structures of *D. magna*, including its shell (see Figure 7a,b), its antennas (Figure 7g,h) and internal structures such as legs and branchias (see Figure 8).

NMs have extremely small sizes, and their interaction with living organisms is a constantly evolving field of research. Studies on *Daphnia* have shown that they can accumulate NMs in their exoskeleton [67,68,69]. The ability of *Daphnia* to remove the NMs attached to its exoskeleton depends on several factors, such as the type of NMs and its concentration [70].

Some studies have shown that *Daphnia* can excrete NMs through its stools or through the hatching of its exoskeleton [22]. However, the elimination capacity may be limited if exposure to NMs is prolonged at high concentrations. It is important to note that research into the effects of NMs on aquatic organisms, such as *Daphnia*, is at an early stage, and further studies are needed to fully understand the long-term effects of exposure to NMs on these organisms [67,68,69,71].

## 3. Materials and Methods

### 3.1. MZ0 Synthesis and Physicochemical Characterization

The MZ0 sample made of maghemite NPs and bulk zeolite 5A was synthesized using a simple stirring and mixing process at room temperature [12]. This method was chosen for its simplicity, efficiency, and scalability. The dried MZ0 sample (powder) was stored in a screw-cap glass container at room temperature. Their detailed physicochemical and magnetic properties are also reported in [12]. The 12 nm maghemite (γ-Fe_2_O_3_) NPs immersed in bulk zeolite 5A have zeta potential and hydrodynamic diameter values of −9.4 mV and 92 nm at neutral pH. Its magnetic properties determined by vibrating sample magnetometry were found to be suitable for magnetic remediation processes (26 emu g^−1^), and they are conserved after kinetic adsorption experiments [12]. The concentration or mass ratio of maghemite and zeolite was below 0.11 (γ-Fe_2_O_3_/zeolite 5A); below this value, the adsorption for lead is remarkable, achieving 100% of removal [12] in real river polluted waters. When exceeding the value of 0.34, the removal percentage decreases, and the system is not effective. Hence, the best system is being explored in this study. It is worth noting that this magnetic fraction retains appropriate magnetic characteristics for magnetic remediation. 

### 3.2. D. magna Culture and Ecotoxicological Assays

*D. magna* cultures were maintained in the wet room at temperatures between 18 and 25 °C, with 16 h of darkness and 8 h of light, and fed with *Scenedesmus* microalgae daily [72].

To start the experiments, adults with eggs were separated in the incubator chamber, and when hatchlings were obtained, they were transferred to a new beaker labeled with the date of birth. After approximately 10 days, the females obtained will begin to give birth to a new litter, and in the third generation, when they show reproductive stability, they will be used for the experiments. Daily monitoring of the culture allows us to identify day one of the newborns, which are separated from the main culture and transferred to a new culture vessel. This way, we can easily track the age of the individuals; they are considered neonates during the first 24 h of life.

### 3.3. Lethal Concentration (LC_50_) Determination

**Scheme 1:**
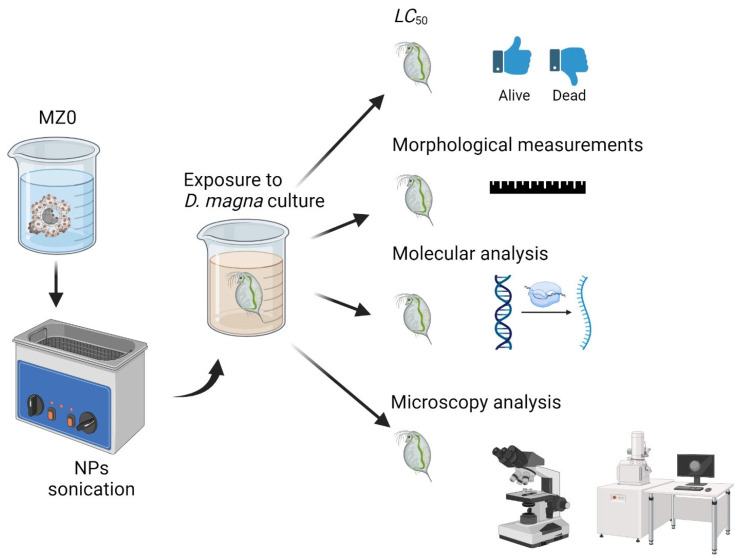
General process of the experimental development of this work, where the sonication process to disperse MZ0 sample is shown, including the exposure of *daphnids* to the MZ0 sample, and each of the procedures to obtain the results, such as the *LC*_50_ values, variation in morphological measurements, molecular analysis to measure gene expression, and analyses conducted using both conventional and electron microscopy.

In this work, a short-term acute assay was developed based on previous literature [15,16]. First, the MZ0 sample was weighed on analytical balance; then, it was transferred to beakers with 200 mL of aquatic medium (where *D. magna* grows) to be sonicated with the digital ultrasonic cleaner (BIOBASE) for 10 min at 40 KHz [30]. See Scheme 1. Second, twenty *D. magna* individuals (neonate and young) were exposed to five different concentrations of MZ0 (25 mg L^−1^, 62.5 mg L^−1^, 125 mg L^−1^, 250 mg L^−1^, and 500 mg L^−1^) for a period of 24 h. The obtained mortalities (%) were converted into Probit numbers that allow predicting the *LC*_50_ concentration [30].

### 3.4. Morphology of Daphnia and Data Analysis

The morphology of *Daphnia* was observed under light optical microscopy before being exposed to various concentrations of the NPs; in the case of exposed neonates, individuals were measured on their first day of life, and after 15 days, the measurements of eye size, antennae, body, heart and caudal spine were performed again with ToupView software v27 x64 3.7.3031. In the case of juvenile individuals, the body size, compound eye, endopod/exopod of the antennae, heart and caudal spine were measured, which are the main morphological parameters for identifying toxicity damage [65].

To determine the significance using the *D. magna* set, a N.C. was employed to conduct an appropriate comparison. The analysis was conducted using the Student’s *t*-test and Wilcoxon’s *t*-test, both with 95% confidence intervals using the software SPSS v27. Beforehand, the Shapiro–Wilk normality test was used to determine whether the data for the morphological parameters were from a normal distribution. The determination of statistical significance, in comparison to the N.C., was made using a *p*-value of 0.05 [15]. The goodness of the linear regression model was validated using the R-squared parameter, R^2^.

### 3.5. Gene Expression by Conventional Electrophoresis Technique

The genes indicated to measure genotoxicity at the expression level are genes related to the development of the compound eye; these genes are *Glass*, *NinaE*, and *Pph13*, whose primers are shown in Table 2. 

First, total RNA extraction was performed by a Genejet Purification Kit from Thermo Scientific (Waltham, MA, USA) and by a reverse PCR Superscript cDNA Synthesis Kit for reverse PCR. The cDNA was amplified using specific primers for *Glass*, *NinaE*, *Pph13*, and Cytochrome oxidase I (COI) as internal control. For quantification, it was subsequently subjected to agarose gel electrophoresis and visualized with Redgel developer in a UV transilluminator to measure band intensity using Gel Analyzer 19.1 software.

To quantify the gene expression of each *D. magna* sample exposed to MZ0, an approximate volume of 250 µL of biological material was taken, consisting of individuals between 24 and 72 h old. After extracting the total RNA and synthesizing the cDNA, PCR was performed, and the use of Image J software 1.54g allowed the quantification of the amplicon according to intensity and band size, identifying each pixel and dark zone of the electrophoretic gel. The statistical analysis was conducted using the Tukey test.

### 3.6. Microscopic Analysis of the Interaction of MZ0 Sample with D. magna

#### 3.6.1. Light Microscopy

Bright-field light microscopy assisted with ToupView software x64 3.7.3031 was used to observe the ecdysis frequency and morphology of *Daphnia* cuticles under different temperature conditions to observe the general morphology of *Daphnia*, including their internal organs and locomotion structures [73,74].

#### 3.6.2. Scanning Electron Microscopy

The FEI brand microscope with the voltage-contrast detector was used to observe the structure of the exoskeleton scales and their arrangement on the body surface. It was also used to analyze the effect of NPs on the morphology of the exoskeleton of *D. magna* [57].

## 4. Conclusions

*D. magna* is more resistant in the neonate stages than in the juvenile stages; despite being smaller, they more effectively resist the conditions of MZ0 in the aqueous environment because of their rapid growth and constant ecdysis. MZ0 can generate toxicity at non-viable concentrations, so it is important to determine the concentrations at which it does not affect this cladoceran. The NMs toxicity in aquatic organisms is a complex phenomenon that depends on several factors, including the age of the individuals, the environmental conditions, the physicochemical composition, the NMs size, the tested concentration, its chemical stability, and others. As evidenced in this study, the exposure of *Daphnia* to the MZ0 hybrid has toxic effects that vary according to the age of the organism, the concentration, and the NMs type. Exposure of neonates to NMs concentrations generates increased body size, while it does not affect antenna size. However, the age of exposure of *D. magna* individuals does matter and is significant, since juvenile individuals showed effects regarding the number of individuals obtained in litters, while neonates recovered after exposure to MZ0, obtaining reproductive rates practically normal for exposure to low concentrations. In both cases, the affected gene is *Glass*, which decreased its expression inversely proportional to the MZ0 concentrations. It has been observed that the heterogeneous characteristics of MZ0 can cause toxic effects in *Daphnia*, including decreased survival, alterations in growth and reproduction, and changes in the morphology and structure of the exoskeleton. It is necessary to continue investigating the exposure of *Daphnia* or other environmental biomarkers to NMs to better understand the mechanisms involved and to establish appropriate preventive measures to minimize the potential risks to aquatic organisms and the environment in general.

## Figures and Tables

**Table 1:** Comparative table of NMs lines and their *LC*_50_, obtained for *D. magna* and other aquatic animals. Notice that the age of individuals was 24 h for all the studies. CeO_2_ indicates cerium dioxide, Au and CuO refers to gold and copper oxide NMs.

Nanomaterial	Specie	Diameter (nm)	*LC*_50_ (mg L^−1^)	Exposure Time (h)	Reference
Ag NPs	*D. magna*	20–30	2.4	48	[17]
CeO_2_ NPs	*D. magna*	5–10	15.8	48	[17]
Au NPs	*D. magna*	21	70	48	[18]
CeO_2_ NPs	*D. magna*	6.5	12	48	[19]
Fe_2_O_3_ NPs	*D. magna*	6	2.4	48	[19]
Ag NPs	*D. magna*	8 ± 0.78	0.03	48	[20]
CuO NPs	*D. magna*	<50	0.15	48	[21]
CuO NPs	*Danio rerio*	-	16.4	96	[22]
Iron oxide NPs	*D. magna*	5–6	82.1	48	[23]
Iron oxide NPs	*D. magna*	<20	654.65	96	[13]
Iron oxide NPs	*Danio rerio*	30	10	96	[24]
Iron oxide NPs	*D. magna*	33.91	50	72	[25]
MZ0	*D. magna*	12 nm for maghemite NPs diameter zeolite 5A (~4 µm)	Neonates 11,314 Young 0.0310	24	This work

**Table 2:** Sequence of the forward (F) and reverse (R) primers of the genes analyzed: *Pph13* (XM_032923697.2), *Glass* (XM_032935199.2) and *NinaE* (XM_032932419.2).

*Pph13*	F	GCTTTGAGACGCAAAGCGGCTATACTGGAA
	R	GGTAACGATGGCTAATTTGCACGGCCTGTC
*Glass*	F	TGGATCCTCATGTGCTTGGTAAGTGTTGAA
	R	CTTATCCTTCTGCGGAAGCTTATGCCATCC
*NinaE*	F	GTCAGCCTTGATGGGCTCGTTGATGCAGAA
	R	TCGGTCGTTACATTCCAGAAGGAATTTTGG

## Data Availability

The original data related to this research can be asked for any time to the corresponding author’s email: juan.ramos5@unmsm.edu.pe.
